# Metrics Used for the Evaluation of Chatbots Providing Cancer Genetic Risk Assessment and Education: Systematic Review

**DOI:** 10.2196/76400

**Published:** 2026-07-15

**Authors:** Jennifer L Scalia, Jessica L Laprise, Jason R Thrift, Christopher L Farrell, Sara M Sarasua

**Affiliations:** 1 School of Nursing, Clemson University, 436 Edwards Hall, Clemson, SC, 29634, United States, 1 401-230-3883; 2 Ambry Genetics, Aliso Viejo, CA, United States

**Keywords:** chatbots, conversational agents, artificial intelligence, metrics, measures, inherited cancer, genetic counseling, genetic risk assessment

## Abstract

**Background:**

Chatbots have recently emerged as an alternative approach for delivering cancer risk assessment and genetic counseling. Understanding the metrics used to describe the user-chatbot experience highlights the strengths and weaknesses of chatbot-assisted health care applications, ensuring safe and reliable medical care. While research supports chatbots in cancer genetic risk assessment and counseling, the evaluation measures remain inconsistent and unsystematic.

**Objective:**

This systematic review analyzes the metrics used to evaluate chatbot platforms providing cancer genetic risk assessment and pretest and posttest genetic education. We examine these measures to identify potential limitations and inform a more systematic evaluative approach.

**Methods:**

A comprehensive search was conducted using PubMed, Web of Science, and Engineering Village. Articles were screened and analyzed using the PRISMA (Preferred Reporting Items for Systematic Reviews and Meta-Analyses) framework. Study and chatbot characteristics were documented, along with variables affecting metric use. Metrics evaluating the user-chatbot experience were extracted, categorized into domains, and organized within the RE-AIM (reach, effectiveness, adoption, implementation, and maintenance) framework to identify assessment gaps and insights regarding application and effectiveness. Risk of bias was assessed using 5 distinct evaluation tools.

**Results:**

This database search retrieved 692 citations, with 14 articles meeting the inclusion criteria. The studies varied in study objective, methodologies, research settings, chatbot functionalities, and participants’ characteristics. A total of 136 measures were extracted and categorized into 16 groups. The number of individual metrics used in each study varied from 3 to 18 (median of 8.5). Measurement groups were organized into 5 domains—user experience, knowledge acquisition, outcomes and behaviors, emotional response, and technical performance—with user experience measures being the most common. Emotional response and technical performance were the least used. Knowledge acquisition measures ranked third and appeared in half of the final study pool. While metrics covered all 5 RE-AIM framework domains, they were unevenly distributed. Risk of bias assessment exposed several study limitations, including small sample size, self-selection bias, and potentially inflated engagement metrics.

**Conclusions:**

This review highlights critical gaps and variability in metrics used to evaluate automated cancer genetic risk assessment and education. Studies most often measured user experience and patient outcomes and behaviors; however, despite its central role in informed consent, knowledge was assessed less consistently and was only moderately ranked. Expanding research efforts and standardizing educational metrics could improve chatbot effectiveness and better support patient decision-making. Important gaps remain in measures of knowledge, emotional response, technical performance, and long-term outcomes, emphasizing the need for increased evaluation in these areas. Using frameworks like RE-AIM can promote comprehensive measurement and a safer and more equitable implementation of novel cancer genetic counseling approaches. Future studies should aim to standardize outcome measures, strengthen missing data methods, and transparently report recruitment and analyses to improve the validity of findings.

## Introduction

### Background

Over the last several decades, automated and artificial intelligence (AI)–enabled technologies have significantly transformed our approach to health care [1]. This transformation is particularly evident in cancer genetics, where chatbots are increasingly used for cancer genetic counseling and risk assessment services [2]. The demand for new automated counseling strategies has developed due to the rapid discovery of inherited cancer genes, leading to a substantial increase in the number of at-risk individuals who need to be identified and offered pretest and posttest cancer genetic counseling and testing [3]. Although there are licensed and certified practitioners trained to provide cancer genetic education and coordinate appropriate testing, the fast pace of scientific discovery has surpassed the ability to train professionals, creating a concerning gap in the health care system [4]. Access to genetic testing and the ability to identify cancer risks that might otherwise go unnoticed can lead to lifesaving strategies, making it essential to improve the delivery of these services [5,6]. Genetic testing has become a vital aspect of precision medicine, significantly prolonging both disease-free and progression-free survival, and resulting in lower mortality rates among patients diagnosed with various types of cancer [7]. As a result, there is a growing demand for innovative and broader-reaching services in this field. However, this shift places the responsibility of patient education and testing on clinicians, many of whom lack the time and training to provide adequate care [8,9]. In response to the rapid evolution of hereditary oncology services, various methods for delivering risk assessment and education are being developed and actively explored [10]. These methods include offering risk assessment and counseling through telehealth or video-based platforms, conducting group counseling sessions, and integrating a genetic professional into specialty clinics to provide point-of-care service [11]. Despite these efforts, the reliance on live genetic providers has created a need for independent solutions. As a result, chatbot technology has emerged as an appealing alternative for delivering information about cancer genetic testing. Genetic counseling chatbots have therefore become a popular solution and are now actively being used in various medical settings [12]. In this review, we focus on chatbot-based and automated conversational tools used to support hereditary cancer risk assessment and genetic education. Although some studies describe these tools as AI-enabled, most interventions included in this review were scripted, rule-based, menu-driven, or natural language processing–assisted chatbots rather than generative AI or large language model systems.

### Chatbots

The use of health care chatbots originated in the early 2000s, primarily using simple rule-based systems designed for basic patient interactions [13]. However, their application in hereditary genetic counseling is relatively recent, with the first publication on this topic appearing only in 2020 [14]. Despite its relatively short history, chatbot-supported hereditary cancer risk assessment and genetic education have gained significant popularity in recent years [14]. These tools can help health care providers identify individuals at risk for genetic predispositions to cancer and offer pretest and, more recently, posttest genetic education [15]. Chatbot technology, commonly accessed via mobile devices or computers, facilitates automated communication and improves access to genetic information and genetic service workflows [16]. This technology enhances the ability to identify at-risk individuals and helps prepare them for genetic testing by delivering text-based information that mimics selected components of conversations traditionally conducted by genetic professionals [17,18].

Genetic testing laboratories serve as the primary providers of mobile counseling options that help health care professionals reduce their workloads while improving patient access to genetic testing [19]. Although most chatbot-based genetic counseling platforms have emerged from these laboratories, trained genetic professionals have typically contributed to their development, and unbiased studies have found these platforms to be well-received and effective [14]. The successful adoption of these tools can be attributed to several factors, including their ability to improve accessibility, optimize resource utilization, and enhance patient engagement [20-22]. Patients particularly appreciate the convenience of mobile apps, which allow them to engage with services at their own pace while maintaining their anonymity [2]. This level of privacy encourages patients to share sensitive information they might otherwise be reluctant to discuss with a human provider [2,23].

Research suggests that chatbots serve as supportive tools that complement the care provided by clinicians, performing simple and often repetitive services [24-26]. This technology enables providers to increase patient volume and focus on more complex cases that require higher levels of expertise [16]. Chatbot-supported pre- and posttest cancer genetic education offers significant advantages for clinicians, especially given recent medical society guidelines that place the responsibility for offering genetic testing on health care providers, who often lack sufficient training in genomics [27]. Consequently, chatbot-based and automated digital tools offer a cost-effective way to improve the accessibility of genetic services [12].

Additionally, chatbots have improved users’ understanding of genetics and have become trusted companions throughout the genetic testing and education process [26]. The broader accessibility is particularly beneficial for individuals in remote or underserved areas, where traditional genetic services may be less available [16,28]. However, despite the positive research findings, the rapid integration of chatbots into oncology practice raises several concerns that require careful examination. For example, users have expressed worries about the accuracy of chatbots [29], issues about privacy and security [30], and doubts regarding their ability to handle complex medical situations given their automated design, which limits them to simpler tasks [31]. These mixed outcomes likely result from the rapid adoption of chatbots and highlight the need for more comprehensive studies to validate their impact. Furthermore, inconsistent measures used to evaluate health care chatbots have hampered efforts to make fair performance comparisons, hindering our understanding of their overall effectiveness [12,32,33]. Therefore, there is a recognized need to standardize and evaluate assessment measures for chatbots early in this process to ensure that research generates accurate and translatable results as the field continues to evolve.

### Assessment Frameworks and Scales

Numerous assessment scales and frameworks have been published to evaluate health care chatbots [32]. A scoping review of 65 studies identified 27 technical metrics assessing chatbots in the health care field. These metrics include usability, comprehensibility, users’ evaluation of the chatbot’s understanding, and visual appearance [32]. The review raises concerns that the lack of standardized metrics could hinder progress in this field and suggests the development of customized evaluation frameworks to ensure successful implementation across various health care specialties [1,32]. To achieve this, researchers must identify the most critical variables relevant to each clinical practice domain [1,34]. This identification process was explored using digital health decision support tools, which are comparable to genetic counseling chatbots, by analyzing the relationships between different variables. The research indicated that user acceptance is a key factor in the successful implementation of chatbot applications [34]. Additionally, several other measurement variables that influence user acceptance were also identified, including ease of use, perceived benefits, and the quality of the service, information, and technology system [34]. Various health care chatbot evaluation frameworks prioritize user acceptance while incorporating these additional variables [1]. However, the application of these frameworks can vary significantly [35], and identifying different study measures can be challenging due to their diverse nomenclature [12,32]. For example, frameworks that have been used to evaluate chatbot systems—such as the Design, Human-AI Interaction, Accessibility, and Feedback framework and the RE-AIM (reach, effectiveness, adoption, implementation, and maintenance) framework—are designed to measure user acceptance. However, the Design, Human-AI Interaction, Accessibility, and Feedback framework’s strength centers on chatbot development, while the RE-AIM framework focuses on population-level outcomes and behaviors [36,37]. Research on digital tools for delivering genetic services emphasizes the importance of establishing a robust evaluative process, such as a framework application that can assess diverse patient populations across various clinical settings [1,10]. Multiple assessment scales have been developed to operate within these frameworks, providing a variety of measures to evaluate the user chatbot experience. Specifically, the Mobile App Rating Scale (MARS), Chatbot Usability Scale (CUS), and System Usability Scale (SUS) are assessment tools that have been successfully used to evaluate digital health tools and chatbot usability within health care research [38-40]. The MARS offers a broader evaluation of mobile apps and is recognized as the first mobile health app rating tool designed to address chatbot quality indicators such as functionality, information quality, and visual appeal [38]. The CUS focuses on evaluating chatbots and includes metrics that assess user-chatbot interaction, trustworthiness, and ease of use. These factors are particularly important in health care settings, such as genetic counseling, because they can significantly influence user behaviors and outcomes [40]. The SUS is a highly adaptable tool that can be applied across diverse digital health care platforms, and because of its ease of use and quick implementation, it is more commonly used when compared to the MARS and CUS [38-40]. This is also evident in chatbot-supported cancer genetics research, where the SUS has been implemented several times to measure chatbot usability among patients with cancer [15,21]. However, it has been recognized that, at times, the intended purpose of assessment scales did not always align with the study’s desired outcomes [10]. This misalignment highlights the importance of understanding the purpose and application of each scale to ensure it aligns with the study’s expected results. Therefore, as digital technology evolves and continues to integrate into different areas of health care, such as hereditary cancer, attention to evaluation scale application and implementing unified measures within a framework may help provide a structured process that ensures planned outcomes are met [32].

### Metrics and Confounding Variables

The evaluation process for chatbot-supported genetic risk assessment and education tools requires focus on both user-centered outcomes and implementation-related outcomes. This involves measuring factors such as effectiveness, adoption, and feasibility in real-world settings [1,12]. Key domains to consider are usability, acceptability, satisfaction, accessibility, completion, efficiency, and perceived usefulness. These criteria are commonly used to evaluate digital and conversational health interventions [32,33]. However, there is often variability in how these domains are defined, measured, and reported in digital health interventions. This inconsistency can limit comparability across studies and complicate the synthesis of evidence [12,32]. In chatbot-based health interventions, the interpretation of evaluation metrics can also be affected by contextual factors such as the target population, clinical setting, chatbot functionality, level of automation, and specific health care tasks being supported [1,33]. To gain a better understanding of the role of chatbots in hereditary cancer risk assessment and genetic education, it is essential to establish clearer and more consistent approaches to metric selection and reporting.

### Objective

This systematic review of the literature analyzed research on patient-facing chatbots used for hereditary cancer risk assessment and education. We systematically extracted and analyzed metrics used to evaluate the chatbot platform and user experience. The identified metrics were organized within a comprehensive evaluation framework to guide the development of a standardized process for assessing the effectiveness of chatbots in this area. Eligible chatbot interventions were limited to scripted, rule-based, menu-driven, or natural language processing–assisted tools. Studies evaluating generative AI or large language model–based chatbots were excluded.

## Methods

### Overview

The systematic analysis was conducted following the PRISMA (Preferred Reporting Items for Systematic Reviews and Meta-Analyses) guidelines to ensure transparency in the reporting of systematic reviews [41].

### Eligibility Criteria

Eligibility criteria for study selection in the literature review were based on the PICOS (participants, interventions, comparison, outcomes, and study design) framework for assessing patients using chatbot-based or automated conversational tools designed to support hereditary cancer risk assessment and counseling (population examined), chatbots (targeted intervention), cancer risk assessment and counseling (intervention purpose), and the measurements used to evaluate chatbot experience (outcomes) [42] (see Table 1). Because there have been limited studies comparing chatbot services to traditional cancer genetic counseling methods, a comparison was not included in the PICOS assessment [14]. We aimed to identify published research investigating how chatbot platforms facilitate cancer genetic hereditary risk assessment and education. Eligibility criteria for publications included (1) chatbots used by patients for hereditary cancer risk assessment and education, hereditary cancer risk, or hereditary cancer genetic education alone; (2) chatbot technology implemented in stand-alone software or web-based platforms, whether rule-based or using natural language processing; (3) men and women over the age of 18; and (4) publications written in English. Exclusion criteria consisted of (1) generative AI/large language model–based chatbots, chatbots using machine learning to remember past interactions, as well as virtual reality or robots that use chatbots as a secondary source; (2) hereditary genetic risk assessment or education unrelated to cancer; (3) hereditary risk assessment or education evaluated outside patient-based settings; and (4) protocol-only publications reporting only planned methodologies without outcome data (see Table 1). Based on the limited scope and novelty of the subject, the search strategy imposed no restrictions beyond protocol-based publications and included all document types to capture relevant data from sources such as abstracts and meeting publications.

**Table 1.** Eligibility criteria (PICOS^a^ framework).

PICOS category	Inclusion criteria	Exclusion criteria
Population	Patients using chatbots Men and women ≥18 years old	Providers/clinicians Not under clinical care Minors (<18 years old)
Intervention	Chatbots delivering hereditary cancer genetic risk assessment and/or education/counseling Rule-based/menu-driven NLP^b^-assisted Stand-alone or web/app-based platforms	Inherited cancer risk/education not addressed Generative artificial intelligence or large language model–based chatbots Contextual bots VR^c^, social robots as primary modality
Comparator	Not applicable	
Outcome	Any measurement of chatbot performance or impact (eg, completion/uptake, satisfaction, usability, knowledge, and decision conflict)	Studies without any evaluative outcome
Study design	Empirical studies (eg, randomized controlled trials, cohort studies, and mixed methods) Full-manuscript publications Abstracts/meeting publications	Protocol-only publications Non-English reported

a PICOS: participants, interventions, comparison, outcomes, and study design.

b NLP: natural language processing.

c VR: virtual reality.

### Information Sources

PubMed, Web of Science, and Engineering Village (Elsevier) were searched to identify relevant studies. Advanced searches were performed in December 2024 and were filtered for publications written in English, retrieved from the years 2010 to 2024. The Web of Science included searching the Web of Science Core Collection and MEDLINE databases, and the Compendex and Inspec databases were searched within Engineering Village.

### Search Strategy

A search strategy for each database was developed that included keywords, subject headings, PubMed Medical Subject Headings terms, and filters to identify eligible studies. The selected search terms were derived from published literature and the guidance of university health science librarians. The search terms, structured from the PICOS framework, focused on chatbots, measurements and assessments, counseling and education, and cancer. Keywords were developed within each concept using Medical Subject Headings terms (when applicable) and subject headings to further widen record retrieval. Search terms were applied to each topic, and the Boolean operator “AND” functioned to connect each search string (Multimedia Appendix 1).

### Data Collection Process

The titles and abstracts were screened based on eligibility criteria, and relevant studies were retrieved in full text and evaluated for inclusion. Two researchers (JLS and SMS) independently reviewed titles/abstracts and full text against the PICOS criteria, as well as the search terms, keywords, and search strategy. Conflicts were resolved with discussion, and if consensus could not be reached, a third reviewer adjudicated. Data from the selected studies were extracted and logged into the developed data retrieval and documentation forms. The first data-charting document included publication characteristics such as first author, year, scientific journal name, study design and setting, chatbot name, and participant type and number (Multimedia Appendix 2 [15,16,21,22,26,43-51]). The second data-charting document includes the user-centric measures applied in each study (Multimedia Appendix 3 [15,16,21,22,26,43-51]). A multistage inductive approach was used to consolidate and synthesize the measures. All reported chatbot evaluation metrics were extracted, coded for overlap, and categorized into 16 thematic measurement groups by two reviewers (JLS and SMS), independently. Study measures included factors such as the time it took the user to complete the chat, the number of users needing to return to the chat for additional information (retention), and whether the user felt the chat helped with test decision-making. Each metric was assigned to only 1 of the 16 measurement groups according to the construct defined by the methodology or measurement tool within each respective study (Multimedia Appendix 3). For example, time-based metrics were classified as engagement unless a study specifically defined them as an outcome or behavior, falling within the “use of information” group. When a metric label conflicted with its construct, it was classified by the construct’s content. In the final stage, these groups were abstracted into 5 high-level conceptual domains representing the core dimensions of chatbot evaluation [32,52]. Discrepancies were discussed and resolved by consensus of two reviewers (JLS and SMS). If consensus could not be reached, a third reviewer adjudicated the disagreement. The final framework was reviewed for coherence and completeness by all researchers.

The 5 metric domains are entitled user experience, knowledge acquisition, outcomes and behaviors, emotional response, and technical performance (Table 2). The user acceptance domain encompasses the measurement groups: ease of use, accessibility, satisfaction, engagement, data confidence, and aesthetics. The knowledge acquisition domain includes 3 groups: retention, knowledge, and effective education. The outcomes and behavior domain covers the impact of genetic testing, medical outcomes, information usage, and user-provider interaction. The emotional response domain focuses on the elements of companionship and feelings of stress or worry, and lastly, the technical performance domain includes only the measurement of technical assistance/data security.

**Table 2.** Five metric domains and their respective measurement groups (number of times each metric group is represented within the respective domain).

Metric domain	Metric groups (count)	Total
User experience	Ease of use (12); accessibility (6); satisfaction (14); engagement (27); aesthetics (3); retention (2)	64
Outcomes and behaviors	Genetic testing impact (23); medical outcomes (5); use of information (13); user-provider interaction (6)	47
Knowledge acquisition	Knowledge (5); effective education (4); data confidence (2)	11
Emotional response	Companionship (5); stress/worry (2)	7
Technical performance	Technical assistance/data security (7)	7

### Quality and Risk of Bias

We evaluated the methodological quality and risk of bias of included studies using design-appropriate evaluation tools: the Critical Appraisal Skills Programme (CASP) checklist for qualitative studies [53], the National Institutes of Health (NIH) Study Quality Assessment Tools for nonrandomized studies (including case series, before-and-after studies, and cross-sectional designs) [54], and the revised Cochrane risk-of-bias tool for randomized trials (RoB 2) [55]. Two reviewers (JLS and SMS) independently assessed each study, recorded their judgments in the respective domains, and resolved any disagreements through consensus. We followed the predefined criteria to rate domain-level judgments: CASP (low concern/some concerns/high concern), NIH (good/fair/poor), and RoB 2 (low risk/some concerns/high risk) [53-55]. When available, related publications or companion methodological reports (eg, protocol papers and related reports) were reviewed to clarify details relevant to the quality assessment. Abstract reports were evaluated but were considered to have a higher risk of bias due to the limited methodological details provided. Because one study included both qualitative and quantitative aspects, each component was appraised separately but counted only once toward the total number of unique studies [43].

## Results

### Study Selection

**Figure 1.**
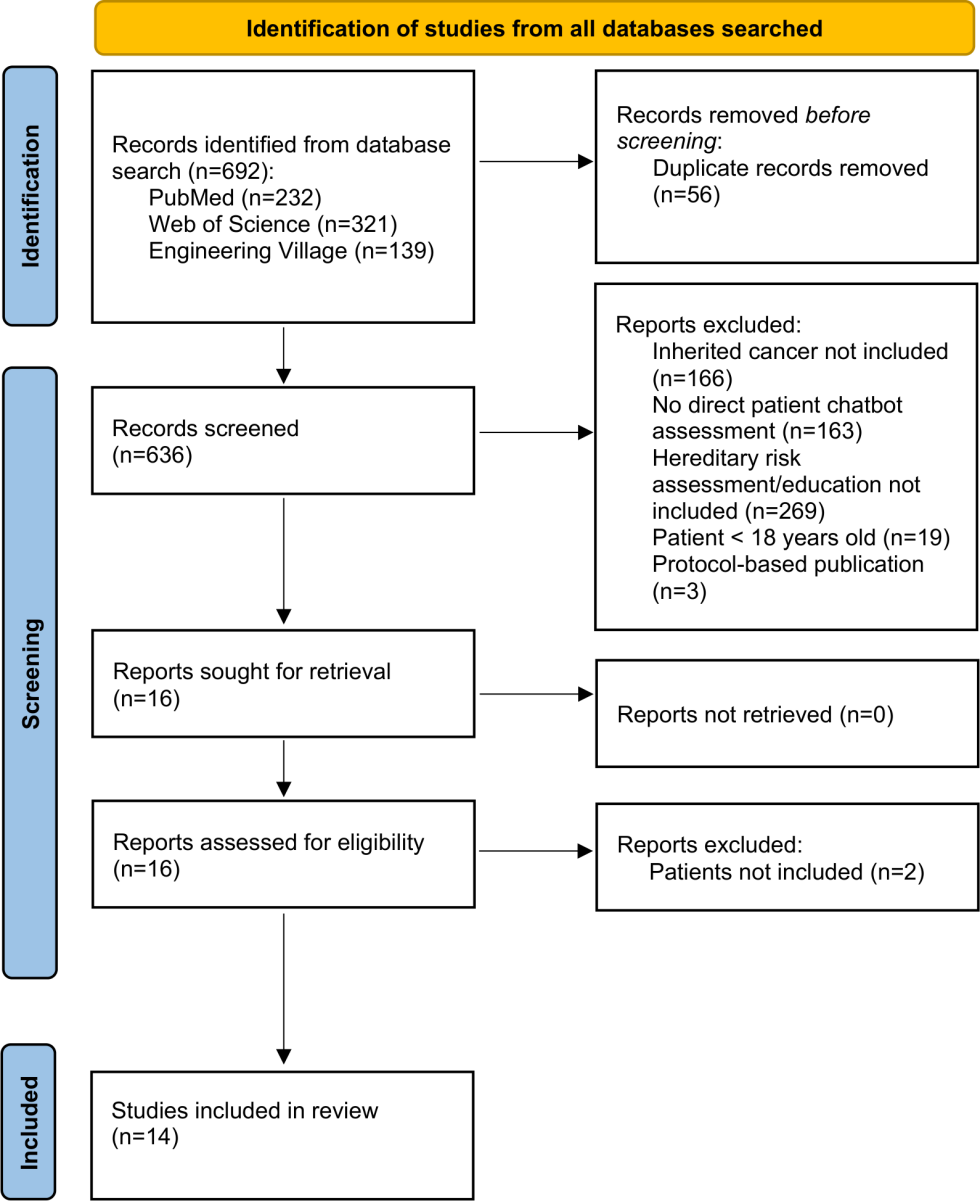
PRISMA flowchart of the study selection process and reasons for exclusion.

The initial literature search yielded 692 records, as illustrated in Figure 1. After removing 56 duplicate studies, 636 records were screened based on titles and abstracts. Of these, 620 studies did not meet eligibility requirements based on reasons detailed in Figure 1. The remaining 16 citations underwent full-text assessment for eligibility. Of these, 2 were excluded, resulting in a final pool of 14 studies that included 11 publications and 3 abstracts.

### Study Characteristics

#### Overview

The study characteristics relevant to this research were extracted from the eligible studies. These include study objective (grouped by similarities), methodology, chatbot name and design, type of patient population, and the number of individuals included (Table 3). Study objectives were composed of four principal aims: (1) conducting comparative-effectiveness trials assessing whether chatbots demonstrate noninferiority to standard of care, (2) delivering pretest education and preparing patients for informed decision-making regarding testing, (3) facilitating risk triage and implementation by systematically identifying eligible individuals and directing appropriate patients toward testing, and (4) evaluating trust, acceptability, and perceived utility to assess whether patients find chatbots trustworthy and usable (Table 3).

**Table 3.** Characteristics of included studies organized by study objective similarities.

Study	Methodology	Objective	Chatbot name	Chatbot function	Participants (sample size)
Comparative effectiveness objective					
Al-Hilli et al [22], 2023	Prospective RCT^a^	Compare chatbot vs in-person counseling	GIA^b^	Pretest counseling	Women newly diagnosed with breast cancer at visit (n=37)
Kaphingst et al [44], 2024	Multisite prospective equivalence RCT	Test equivalence of chatbots vs SOC^c^	GIA	Pretest counseling	Primary care patients eligible for genetics (n=3073)
Pretest education/decision preparation objective					
Chavez-Yenter et al [45], 2021	Feasibility; descriptive analysis	Describe patient-educational chatbot interactions	GIA	Pretest counseling	Primary care clinic patients (n=103)
Rupert et al [46], 2013	Feasibility clinic pilot	Assess chatbot to improve education and decision-support	Cancer in the Family	Risk and pretest counseling	Routine provider clinic, scheduled visit (n=48)
Soley et al [15], 2023	Feasibility pilot	Assess chatbot education and testing decisions/adoption	GIA	Pretest and posttest counseling	Patients with pancreatic cancer prior to visit (n=60)
Visvanathan et al [21], 2023	Prospective single-center feasibility pilot	Assess chatbot education, usability, and adoption	HealthFAX platform	Pretest counseling	Patients in active treatment for breast or prostate cancer (n=51)
Risk triage/implementation					
Dohany et al [47]^d^, 2020	Multisite prospective	Use chatbot to identify at-risk patients	CARE^e^	Risk	Ob/Gyn clinic prior to visit (~9750)
Heald et al [16], 2021	Feasibility; prospective cohort	Identify at-risk patients and delivering education	Not specified	Risk and pretest counseling	Patients scheduled for colonoscopy (n=487)
Maisenbacher et al [48]^d^, 2021	Descriptive cross-sectional	Test chatbot to identify at-risk patients	NEVA^f^	Risk	Patients contacted via email (n=157)
Monsour et al [49]^d^, 2022	Retrospective cohort	Identify at-risk patients and educate; order testing	CARE	Risk and pretest counseling	Rural GI^g^ clinic, routine visit (n=1029)
Nazareth et al [50], 2021	Multicenter retrospective observational	Identify at-risk patients, educate, and route to test	GIA	Risk and pretest counseling	Women’s health clinics before scheduled visit (n=61,070)
Sato et al [43], 2024	Feasibility study; mixed methods	Validate chatbot risk screening and assess usability	Unspecified-using IBM platform	Risk	Predominantly women with cancer, scheduled visit (n=11)
Trust and usability					
Schmidlen et al [51], 2019	Qualitative focus groups	Assess chatbot accessibility/usability	GIA	Pretest counseling	Research participants enrolled in a prior study (n=62)
Siglen et al [26], 2023	Qualitative interviews	Explore perceived utility, trust, and impact	Rosa	Pretest counseling	Women at risk for breast/ovary cancer, scheduled visit (n=16)

a RCT: randomized controlled trial.

b GIA: Genetic Information Assistant.

c SOC: standard of care.

d Abstract only.

e CARE: Comprehensive Assessment, Risk, and Education.

f NEVA: Natera’s Educational Virtual Assistant.

g GI: gastroenterology.

#### Study Objective

Across the 4 study objective groups, 2 comparative effectiveness trials evaluated whether chatbot pretest services matched the standard of care, using noninferiority measures for knowledge, satisfaction, and service completion [22,44]. A broader group of studies focused on risk triage and implementation, identifying eligible patients and streamlining next steps, with an emphasis on engagement and clinical accuracy [16,43,47-50]. Another set of studies explored pretest education and decision preparation by delivering education outside of clinic times and enhancing patient decision-making through chatbot engagement and usability [15,21,45,46]. Lastly, 2 qualitative studies examined themes of trust, acceptability, and perceived utility through interviews and focus groups, informing the deployment of chatbots [26,51].

#### Methodologies of Reviewed Studies

The study methods included randomized controlled trials (RCTs), prospective and retrospective observational designs, feasibility studies, mixed methods research, qualitative studies, and abstract-only reports. Two studies were RCTs [22,44]. Six studies used prospective feasibility, pilot, or descriptive designs [15,16,21,43,45,46], including one mixed methods study [43]. Two studies used qualitative approaches, including interviews or focus groups [26,51]. One study was a large multicenter retrospective observational cohort study [50]. Three studies were abstract-only implementation or descriptive reports [47-49].

#### Chatbot Name

The Genetic Information Assistant chatbot was the most commonly studied (n=6, 43%) [15,22,44,45,50,51]. The second most popular was the Comprehensive Assessment, Risk, and Education (n=2, 14%) platform [47,49], and the remaining studies examined a different conversational agent. These included Natera’s Educational Virtual Assistant [48], 3 novel chatbots developed by the author’s institution (Cancer in the Family, Rosa, and HealthFAX platform) [21,26,46], 1 unnamed platform using IBM’s cognitive computing service [43], and 1 platform that was not specified [16].

#### Chatbot Purpose

The chatbot and automated conversational tools were grouped into four service categories: (1) obtaining cancer family history for the calculation of the user’s chance of carrying a genetic variant increasing cancer risk, (2) providing pretest cancer genetic education, (3) offering both risk assessment and cancer genetic education, and (4) providing pre- and posttest cancer genetic education. Four (29%) chatbot systems offered risk assessment and counseling [16,46,49,50], and 3 (21%) chatbots assessed hereditary risk only [43,47,48]. Six (43%) chatbots were designed to provide pretest counseling [21,22,26,44,45,51], while only 1 (7%) offered pre- and posttest education [15].

#### Study Participants

Most populations studied were unaffected by cancer (n=10, 71%) [16,26,44-51], and were recruited during scheduled clinic visits (n=9, 64%) [15,16,22,26,43,46,47,49,50]. One population was explicitly recruited from a patient population scheduled for a colonoscopy procedure [16]. Four (29%) studies examined individuals already diagnosed with cancer, including women diagnosed with early-stage breast cancer [22], a predominantly female cohort with cancer of unspecified type [43], men and women diagnosed with pancreatic cancer [15], and women and men undergoing active treatment for either breast or prostate cancer [21]. The number of research participants in each ranged from 11 to 61,070, with a median of 82.5 participants per study across the 14 publications. Nine studies enrolled fewer than 200 participants [15,21,22,26,43,45,46,48,51], in contrast to the 3 largest studies enrolling approximately 3000, 10,000, and 61,000 participants [44,47,50].

### Study Metrics and Metric Domains

Among the 14 eligible studies, 136 measures were used (Multimedia Appendix 3). The 136 measures extracted included repetitive and overlapping measures and, as a result, were grouped into 16 measurement categories, which included engagement, ease of use, use of information, satisfaction, genetic testing impact, effective education, knowledge, user-provider interaction, companionship, medical outcomes, retention, technical assistance/data security, accessibility, data confidence, stress/worry, and aesthetics. The number of individual metrics used in each study varied from 3 to 18, with a median of 8.5 (Table 4). The 16 measurement groups were then categorized into 5 metric domains: user experience—evaluating how users engage with the chatbot; knowledge acquisition—assessing the degree to which users improve their understanding of genetic information and their implications; outcomes and behaviors—related to health outcomes, behavior changes, and decision-making; emotional response—documenting users' emotional reactions; and technical performance—evaluating the chatbot’s response time, error rates, security, and overall system reliability (Table 2).

**Table 4.** Individual metrics and measurement groups by study.

Study	Individual metrics, n	Measurement groups represented (individual metrics within group, n)
Al-Hilli et al [22], 2023	6	Satisfaction (1), knowledge (1), genetic testing impact (2), medical outcomes (2)
Chavez-Yenter et al [45], 2021	9	Engagement (3), satisfaction (2), genetic testing impact (3), use of information (1)
Dohany et al [47],^a^ 2020	4	Engagement (2), satisfaction (1), genetic testing impact (1)
Heald et al [16], 2021	8	Engagement (2), genetic testing impact (5), user-provider interaction (1)
Kaphingst et al [44], 2024	3	Engagement (2), genetic testing impact (1)
Maisenbacher et al [48],^a^ 2021	3	Engagement (1), genetic testing impact (2)
Monsour et al [49],^a^ 2022	6	Engagement (1), genetic testing impact (3), medical outcomes (2)
Nazareth et al [50], 2021	11	Engagement (5), satisfaction (1), retention (1), genetic testing impact (3), technical assistance/data security (1)
Rupert et al [46], 2013	13	Engagement (1), ease of use (1), satisfaction (1), knowledge (1), effective education (1), use of information (3), user-provider interaction (2), medical outcomes (1), companionship (1), stress/worry (1)
Sato et al [43], 2024	18	Engagement (2), satisfaction (1), ease of use (3), accessibility (3), data confidence (1), aesthetics (2), use of information (2), user-provider interaction (1), technical assistance/data security (3)
Schmidlen et al [51], 2019	8	Satisfaction (3), ease of use (1), accessibility (1), effective education (1), use of information (1), technical assistance/data security (1)
Siglen et al [26], 2023	18	Engagement (4), satisfaction (2), ease of use (1), accessibility (1), knowledge (1), effective education (1), use of information (3), companionship (3), stress/worry (1), technical assistance/data security (1)
Soley et al [15], 2023	17	Engagement (3), satisfaction (1), ease of use (3), knowledge (2), effective education (1), genetic testing impact (2), use of information (2), user-provider interaction (2), technical assistance/data security (1)
Visvanathan et al [21], 2023	12	Engagement (1), satisfaction (1), ease of use (3), accessibility (1), data confidence (1), aesthetics (1), retention (1), genetic testing impact (1), use of information (1), companionship (1)

a Abstract only.

### Narrative Synthesis

#### Synthesis of Study Characteristics

Across studies, outcome measures clustered into four objective categories: (1) comparative effectiveness, (2) risk triage/implementation, (3) pretest education/decision preparation, and (4) trust/usability (Table 3). Comparative effectiveness objectives primarily used metrics related to clinical equivalence and uptake (ie, completion rates and time to treatment) [22,44]. Risk triage and implementation studies emphasized throughput and yield measures, including completion percentages, rate of meeting National Comprehensive Cancer Network criteria, and order volumes, alongside workflow-related indicators such as electronic medical record integration, patient engagement, and sustainability [16,43,47-50]. In contrast, pretest education and decision preparation studies most often measured knowledge change, decision readiness, discussion/referral prompts, and time to complete educational content [15,21,45,46]. Trust and usability studies used qualitative themes and quantitative usability/satisfaction measures, including assessment of emotional safety (ie, reports of increased worry) and the frequency of human counselor assistance [26,51].

Methodological approaches spanned RCTs [22,44], prospective feasibility, pilot, or descriptive designs [15,16,21,43,45,46], qualitative studies using thematic or semistructured interviews and focus group assessment [26,51], one large multicenter retrospective observational cohort analysis [50], and abstract-only reports [47-49]. Across this literature, feasibility and early-stage evaluations were most common, with a total of 6 studies [15,16,21,43,45,46], and a substantial proportion of studies evaluated the same chatbot platform named Genetic Information Assistant [15,22,44,45,50,51] (Table 3). This commonality enhanced comparability for specific outcomes but limits diversity in chatbot functionalities and associated measures. Across study populations, most cohorts consisted of unaffected patients [16,26,44-51], with fewer studies focused on patients already diagnosed with cancer [15,21,22,43] (Table 3). Sample sizes were highly variable, with many small studies and limited large-scale research (Table 3). Abstract-only publications constrained by word count primarily emphasized key evaluation measures [47-49], while full-length peer-reviewed articles included a wider variety of metrics [15,16,21,22,26,43-46,50,51] (Table 3).

#### Synthesis of Metric Domains

Across the 5 metric domains (Table 5), measures most frequently fell within the user experience category (64/136 measures, 47%), and this domain was used by all 14 studies. Outcome and behavior measures represented the second most prevalent category (47/136, 35%) and were also represented across all 14 studies. By comparison, knowledge acquisition measures were used less often (11/136, 8%) and appeared in half of the total studies (7/14, 50%). Emotional response (3/14, 21%) and technical performance (5/14, 36%) domains were evaluated in fewer studies, each comprising only a small fraction of total measures (7/136, 5%), with companionship and technical assistance/data security representing the most commonly used groups within those domains (Table 2).

**Table 5.** Distribution of evaluation measures and included studies by metric domain.

Metric domain	Studies using domain, n	Measures used within domain (N=136), n (%)
User experience	14	64 (47)
Outcomes and behaviors	14	47 (35)
Knowledge acquisition	7	11 (8)
Technical performance	5	7 (5)
Emotional response	3	7 (5)

### Quality and Risk of Bias

The CASP-based quality assessments indicated that the qualitative reports (n=3) were rated as having “some concerns” [53]. This was primarily due to small sample sizes, participation and data collection constraints, and limited reporting regarding the researcher’s reflection on their role [26,43,51]. Among the nonrandomized studies evaluated for methodological quality using NIH tools [54] (10 records contributing to quality assessment), the ratings included “good” for 2 studies (both case series) [16,21], “fair” for 4 studies (which included one before-and-after study without a control group and several observational or cross-sectional studies) [15,43,46,50], and “poor” for 4 studies (mostly feasibility or abstract-only reports) [45,47-49]. Both RCTs (n=2) received ratings of “some concerns” according to RoB 2 [55], due to practical limitations regarding blinding, possible deviations from the intended interventions, and issues related to selective outcome reporting and data completeness [22,44]. Multimedia Appendix 4 provides study-specific ratings along with the evaluation tool’s domains [15,16,21,22,26,43-51]. A dagger (†) indicates studies where there was an initial discrepancy between independent reviewers (JLS and SMS) that was resolved through consensus, with the final rating reported. Additionally, the study by Sato et al [43] reports both qualitative interviews and quantitative observational assessments, but it counted only once among the 14 studies.

### Mapping Extracted Measures to an Evaluative Framework

Literature suggests that evaluation measures for chatbot platforms are often inconsistent and influenced by confounding factors [12,32,33]. Therefore, a standardized framework or “roadmap” is needed to guide the use of these metrics. Understanding if these novel automated health services are being implemented as planned and producing the desired health care outcomes requires consistent and effective evaluation techniques [20]. Therefore, a framework such as RE-AIM [37] could structure a systematic evaluation process to ensure safe and reliable patient care while highlighting important measures that may be underused or excluded [29,46]. Although various frameworks are designed to assess digital health care services, RE-AIM explores the impact on the user and is deemed optimal for real-world situations [34,56], thus aligning with the user-patient genetic counseling experience. The RE-AIM framework is a comprehensive approach to evaluate health interventions with a focus on 5 dimensions: the impact on the user, specifically who is being identified or engaged (reach), outcomes such as the completion of testing, knowledge or intent to change behavior (effectiveness), user acceptability and willingness to engage with the intervention (adoption), the consistency across users and settings including considerations of feasibility, time, workflow and usability (implementation), as well as the long-term sustainability of the system (maintenance) [37]. This framework has been successfully applied to health care chatbot evaluation, including research examining user acceptance of conversational agents supplying information about autoimmune disease and knowledge related to the COVID-19 vaccination in Southeast Asia [57,58].

**Figure 2.**
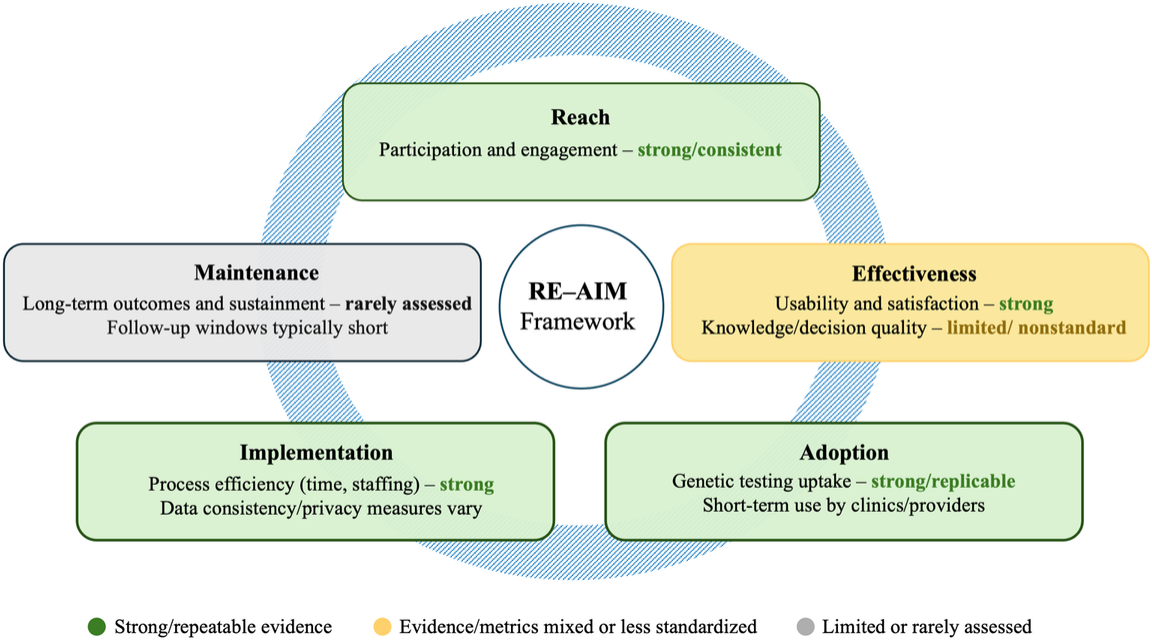
Metrics from the included studies were mapped onto the 5 dimensions of the RE-AIM (reach, effectiveness, adoption, implementation, and maintenance) framework.

When applying the extracted measures from this systematic review to the 5 dimensions of the RE-AIM framework, it is evident that the measurements reach all 5 areas of this paradigm; however, their distribution is uneven (Figure 2). The dimensions of reach, implementation, and adoption, along with usability and satisfaction in the effectiveness dimension, provide the strongest and most consistent evidence of measurement use. Nazareth et al [50] and Dohany et al [47] demonstrate large-scale real-world settings using multisite chatbot invitations and engagement measures that support the reach dimension. In contrast, Heald et al [16] and Chavez-Yenter et al [45] examine detailed processes within clinic-based workflows, focusing on the implementation of these recruitment strategies. There is further evidence supporting implementation as well as the domain of adoption, especially regarding testing uptake and measures of process efficiency. This is highlighted by the high percentage of high-risk patients who were tested after the introduction of chatbots, with reports indicating uptake rates of 71% [16] and 86% [21]. Additionally, metrics used that are related to time and streamlined workflows underscore the measurement of efficiency gains [16,50]. However, while these efficiency improvements were evident, the resulting literature focused on patients and clinics that used chatbots for only a short period of time [21,43,46]. Moreover, the implementation of data consistency and privacy measures varied across the 14 studies examined. Privacy measures ranged from formal compliance certifications like the Health Insurance Portability and Accountability Act to anticipated security practices, with some studies providing limited to no reporting on these issues [50,51]. Data consistency also fluctuated due to privacy constraints, differences in electronic medical record integration, and the availability of data [21,26,50,51]. Usability measures primarily fell within the effectiveness dimension, evidenced by qualitative endorsements, high satisfaction metrics, and ratings that reflect ease of use and time-savings [21,26]. However, the measures of knowledge and decision quality within this domain are limited and less standardized, with fewer studies using validated scales. Only Al-Hilli et al [22] use clearly validated knowledge and satisfaction scales, while other researchers focused on decision intentions and self-reported metrics rather than validated decisional outcomes [45,46]. Finally, the evaluation of long-term maintenance and sustainability was noted to be rare, specifically beyond short follow-up periods. This is evident in the studies conducted by Rupert et al [46] and Visvanathan et al [21], which report follow-up durations of only 8 weeks and 6 weeks, respectively. Both studies acknowledged that these timeframes are insufficient for assessing long-term outcomes. Additionally, the small sample size and limited short-term usability and accuracy check in Sato et al [43] Japanese feasibility study emphasize the limitations in evaluating long-term sustainability.

## Discussion

### Principal Findings

In this systematic review, we examined how researchers measure the effectiveness of chatbots used for cancer genetic risk assessment and education. Our findings revealed that user experience was the most used metric, while emotional response and technical performance measures were applied the least (Table 5). This aligns with previous research indicating that user experience is a key metric for health care chatbot assessment [1]. User experiences significantly influence usability, trust, and engagement, and are also a relatively quick and cost-effective measure to implement [26,33,44,51]. The limited use of emotional response and technical performance metrics is consistent with findings from other studies evaluating the assessment methods for digital genetic services [10,44,59]. Despite the increasing research into genetic counseling and digital tools, the understanding of emotional response remains significantly underexplored, which could greatly enhance automated genetic counseling [60]. This gap may stem from the limited ability of most chatbot platforms to convey empathy effectively [44,59]. Outcomes and behavior measures were the second most frequently used domain in our study, tied for first in the number of studies that incorporated them (Table 5). Since the goal of improving patient care is essential in the development of health care chatbots, this observation is unsurprising [61]. This domain plays a critical role in evaluating the overall impact of technology on health care services and medical care [62]. In contrast, an important insight from our analysis is the inadequate emphasis on knowledge acquisition measures (Table 5). Chatbot platforms have been developed in this field to assist providers in obtaining informed consent for genetic testing. This process fundamentally relies on effective patient education and comprehension [63]. While acquiring knowledge is only one component of the genetic counseling process, it is a vital part, as informed consent standards mandate that patients receive sufficient information and understand essential details before deciding on genetic testing [63]. Although psychosocial support and other elements of counseling play significant roles in cancer genetic education, they are secondary in determining whether the consent provided is genuinely informed [63,64]. The educational content that needs to be communicated to patients before and after cancer genetic testing is governed by complex national guidelines [65]. If chatbots fail to deliver this information effectively, they will not achieve their primary function of addressing the knowledge gap between health care providers and patients [9,65]. As a result, this study emphasizes the importance of improving measures and incorporating tools to evaluate the type and amount of genetic knowledge that users gain from virtual agents. This evaluation is vital for validating the role of these agents in fairly disseminating information related to genetic test decision-making. Additionally, our study highlights the need for standardized scales or clearly defined measurement approaches to improve comparability across chatbot studies [32]. Research on genetic chatbots will require various evaluation tools tailored to specific factors such as study objectives and methodologies. Therefore, it is essential to establish a set of scales that cover the key elements of a comprehensive evaluation. This approach can provide researchers with a range of measurement options tailored to their specific needs [1,29]. Established tools such as the SUS for evaluating digital system usability and the KnowGene Scale for measuring cancer genetic testing knowledge are both valid and reliable, even though they assess different constructs [39,66]. These scales have been used for assessments in both automated and traditional cancer genetic counseling, making them valuable for future research [21,66]. However, identifying appropriate scales for evaluating specific measures within a given field of study can be challenging and may require adjustments to existing tools to achieve desired outcomes. For example, the 16-item KnowGene Scale includes language relevant to a live clinician providing education, which may not apply to questions directed at a user receiving education from an automated device [66].

This review also analyzed study-based characteristics, revealing that the primary driver of metric selection and utilization was the study objective. The objectives determined which constructs and datasets needed to be measured, guiding the selection of the most appropriate metrics. However, several additional confounding variables were identified that influenced metric utilization across studies. These variables included the scope and depth of the evaluation (eg, methodological approach), the operationalization of measures within the chatbot (eg, chatbot design and implementation), and contextual constraints that impacted objective-driven measurement choices (eg, research setting and participant population). For example, in the ENGAGE study, which enrolled cancer patients undergoing active treatment who had already received testing through nongenetic providers, the primary focus was on adoption and user experience rather than risk-screening outputs, such as patients meeting test criteria or test uptake [21]. All patients chose to complete the chatbot at home, and the reported metrics included chatbot access and opening rates, time on task, completion rates of the educational content, as well as indicators of usability and satisfaction [21]. In contrast, studies designed to support hereditary cancer risk assessment or testing triage more commonly reported screening completion, testing eligibility, genetic testing uptake, and pathogenic variant detection [16,47,49,50]. These differences suggest that metric selection was shaped not only by the overall goal of evaluating chatbot effectiveness, but also by the clinical function of the chatbot and the setting in which it was deployed. Although the study objective was a significant factor in metric selection, the research methodology also played an important role. Methodological approaches not only predicted how measurements were taken but also influenced the perceived necessity of those measurements. Lastly, the inherent constraints of abstract publication limited both the timeframe and the number of metrics used and/or reported. While factors such as publication type and research setting may appear intuitive, they underscore important considerations for developing an effective assessment framework [1].

It is also important to recognize that the ease or difficulty of implementing these measures can impact metric utilization. For example, certain metrics, such as session duration and completion, are easier to implement because they involve data that can be directly obtained from standard platform telemetry. In contrast, constructs such as knowledge acquisition and emotional impact are related to internal states and learning, which cannot be reliably inferred from behavior alone [66,67]. These outcomes may require validated instruments, participant-reported measures, or follow-up assessments, which can increase study burden and may explain why they were less frequently reported [66,67]. Therefore, this review highlights the significant impact of various determinants on the evaluation metrics of chatbot performance, underscoring the challenges associated with developing a robust and reliable assessment framework [32].

Obtaining consistent measures from unified assessment scales positioned within a structured evaluation framework, like RE-AIM, can promote equitable metric distribution in genetic research, further ensuring safe and reliable medical practices [29,56]. By mapping the metrics extracted from the 14 studies examined onto the RE-AIM framework, we confirmed that these measures reached all 5 framework domains. However, evidence suggests that certain measurement areas were less standardized, limited, and, at times, rarely assessed (Figure 2). Metrics within the reach and effectiveness domains were strong, supported by large screening and participation numbers [16,47,48,50], as well as sound evidence for service completion and usability [16,44]. Adoption and most implementation measures were similarly strong [26,43,45,51]. However, implementation metrics related to data consistency were variable, raising concerns that the assessment of equitable use may not be adequately captured. Equitable distribution of educational content is a critical functional aspect of chatbots and plays a vital role in the informed consent process. The fair dissemination of information is essential for empowering patients to make well-informed decisions about cancer genetic testing [1]. Although the functionality of these chatbot systems permits users to navigate freely within the platform, it does not ensure patients receive the required education needed to make informed decisions about genetic testing [26,43,45,51,68]. Consequently, this increases the responsibility of ensuring informed consent requirements on the untrained health care provider [8,9]. Therefore, this measurement variability exposed by the RE-AIM framework highlights the need for a more uniform measurement process to ensure that we respect patients’ rights regarding consent [64].

Measures of knowledge gains could also serve as a measure for evaluating the distribution of educational information. Unfortunately, the RE-AIM framework shows that measures of knowledge acquisition are limited and less standardized (Figure 2). Although acquiring measures of knowledge acquisition can be more challenging, this recognized gap, indicated by both the application of RE-AIM and the analysis of study metrics, underscores the necessity of incorporating this domain as a primary research objective to ensure chatbots in this space are delivering safe and equitable patient care. The dimension of maintenance was rarely critically assessed, potentially impacting several areas of care, including medical outcome validation and quality of educational content. Nevertheless, the novelty of this field could be contributing to the metric gap observed in this framework domain.

In summary, this review evaluates various measures currently used in research to assess the effectiveness of chatbots in cancer genetic risk assessment and counseling. It identifies 5 metric domains, with user experience being the most frequently measured. In contrast, emotional response and technical performance metrics were less commonly used, revealing a significant gap in the evaluation of these factors. Additionally, measures of knowledge acquisition were also underrepresented, despite their critical role in informed consent and patient education. The study highlights the need for standardized evaluation scales to facilitate fair comparisons of chatbot performances. It also identifies variables, including study objectives and chatbot design, that can influence these metrics. Lastly, the RE-AIM framework was used to map the extracted measures, revealing important gaps in assessment, including the limited measures of knowledge and the lack of long-term outcome metrics. Overall, these findings underscore the need to refine measurement tools and applications to validate the role of chatbots in effectively disseminating genetic information.

### Strength of Evidence

The overall quality of evidence is mixed. Qualitative studies generally meet their aims but often lack complete reporting on aspects including sampling rationale, the role of the researcher, and how deeply they analyze their data [26,51]. These gaps can affect the trustworthiness and usefulness of study results. In nonrandomized studies, common issues included selection bias, lack of comparison groups, and uncontrolled confounders [16,45,46,50,69]. These problems can weaken the conclusions about chatbot effectiveness in improving outcomes like knowledge and decision-making. Case series studies with clear outcomes show that chatbots can be feasible and acceptable [16,21]. However, their single-arm design limits generalizability [21]. Although the 3 abstract reports provide valuable outcomes, their limited reporting of methodology results in a high risk of bias [47-49]. The 2 RCTs reduced the overall risk of bias rating; however, they still demonstrated concerns commonly seen, such as difficulties with blinding and some uncertainties about how outcomes are measured [22,44,70]. These findings suggest that while these are encouraging results, they should be interpreted cautiously. Future studies could reduce the risk of bias by using standardized outcome measures, managing missing data more effectively, and being more transparent when reporting recruitment and their analysis processes, which could strengthen the validity of results.

### Limitations

This systematic review had several limitations. First, due to the field’s novelty, a limited amount of research met eligibility requirements. As a result, more extensive studies should be conducted to confirm the outcomes reported in this study. Four of the examined studies were authored by employees of the genetic testing laboratories that designed the respective chatbot technology [47-50]. This relationship may introduce biases about the chosen measurements for evaluating these chatbot systems. Additionally, because the laboratories determine the chatbot metrics tracked at a broad system level, this can potentially limit or promote the reporting of specific measures in the research on this topic. Another limitation is that several included studies were single-arm feasibility designs or abstracts with limited methodological detail, elevating the risk of bias and reducing external validity (Multimedia Appendix 4). Lastly, despite efforts by authors to minimize bias, some degree of subjective judgment remains possible when categorizing and synthesizing the extracted measurements from the studies.

### Conclusions

Measures extracted from the 14 studies were placed in 5 high-level domains: user experience, outcomes and behaviors, knowledge acquisition, emotional response, and technical performance. Measures of knowledge acquisition were found to be limited and unstandardized, underscoring their importance in informed consent and patient safety. The study advocates for standardized evaluation scales to improve assessments and enable fair comparisons between chatbot performances. While the measures covered all 5 dimensions of the RE-AIM framework, they were unevenly distributed, revealing gaps in long-term sustainability, data consistency, and knowledge. Lastly, variables such as study objectives and chatbot designs can affect measurement effectiveness, necessitating careful selection during evaluation. In conclusion, this review exposes critical gaps in automated genetic risk assessment and counseling metrics and proposes a more structured evaluation process to ensure safe, equitable, and effective implementation of this technology, while stressing the need to conduct more rigorous metric-focused research to validate these findings and improve future study designs.

## Supplementary material

10.2196/76400 Multimedia Appendix 1 Search strategy for each database.

10.2196/76400 Multimedia Appendix 2 Study data extraction form.

10.2196/76400 Multimedia Appendix 3 Extracted study metrics grouped into measurement categories and organized into metric domains.

10.2196/76400 Multimedia Appendix 4 Risk of bias tool, rating assigned, and rationale for each study.

10.2196/76400 Checklist 1 PRISMA 2020 checklist.

## Abbreviations

AI: artificial intelligence
CASP: Critical Appraisal Skills Programme
CUS: Chatbot Usability Scale
MARS: Mobile App Rating Scale
NIH: National Institutes of Health
PICOS: participants, interventions, comparison, outcomes, and study design
PRISMA: Preferred Reporting Items for Systematic Reviews and Meta-Analyses
RCT: randomized controlled trial
RE-AIM: reach, effectiveness, adoption, implementation, and maintenance
RoB 2: revised Cochrane risk-of-bias tool for randomized trials
SUS: System Usability Scale
