# Supplementary material for: Metrics Used for the Evaluation of Chatbots Providing Cancer Genetic Risk Assessment and Education: Systematic Review
Source: JMIR AI. 2026 Jul 15;5:e76400. doi: 10.2196/76400 (PMC13372268; doi:10.2196/76400)
Supplement: Multimedia Appendix 4 [file ai-v5-e76400-s004.docx]

**Multimedia Appendix** Scalia et al., 2026

Risk-of-Bias Assessment for Included Studies

| Study | Tool | Overall rating | Rationale |
| --- | --- | --- | --- |
| Schmidlen 2019 [51] | CASP (qualitative) | Some concerns | Clear aims and an appropriate qualitative focus group design; data collection, transcription, coding, and consensus analysis described. A related cascade-testing implementation paper [2] provided context on later deployment of one chatbot component but did not materially alter the qualitative quality assessment. However, transferability was limited by recruitment from a single health system and a largely older, White sample already enrolled in a genomic research cohort. Reflexivity was limited, and thematic saturation may not have been reached. |
| Siglen 2023 [26] | CASP (qualitative) | Some concerns | Clear aims and appropriate qualitative interview design; recruitment, interview procedures, transcription, and stepwise-deductive inductive analysis were well described. A related development paper [4] provided useful context on Rosa chatbot’s participatory, iterative development and usability testing. However, the evaluation relied on a small sample from consenting Norwegian-speaking chatbot users, and individual chatbot-use data were unavailable. |
| Sato 2024 [43] (qualitative component) | CASP (qualitative) | Some concerns | Clear aims and an appropriate pilot feasibility design; chatbot completion, questionnaire procedures, post-use interviews, transcription, and descriptive coding reported. A related development paper [6] provided context on prior scenario-based testing and expert review of the risk-screening chatbot. However, the qualitative component was limited by a very small single-center sample, all-female and highly educated participants, limited reflexivity, no discussion of saturation, and brief descriptive qualitative analysis. |
| Rupert 2013 [46] | NIH Before–After (Pre-Post) Studies With No Control Group | Fair | Clear objective prespecified eligibility criteria, a well described intervention, and appropriate pre/post outcome assessment for a pilot evaluation. However, confidence is limited by the small, selected sample, homogenous patient population, lack of control group, attrition, short follow-up, and reliance on several self-reported outcomes. The findings support feasibility and short-term knowledge/risk communication effects without strong conclusions about effectiveness or generalizability. |
| Visvanathan 2023 [21]† | NIH Case Series | Good | Clear objective, defined eligibility criteria and study population, detailed automated education intervention description, relevant feasibility and outcome measures, and transparent reporting of results. Limitations included the small single arm pilot design, single-center setting, unclear consecutive recruitment, absence of a control group, and short follow-up which were considered when interpreting the findings. Findings support feasibility and acceptability among users. |
| Heald 2021 [16]† | NIH Case Series | Good | Clear objective, defined eligible population, well-described intervention, relevant uptake and implementation outcomes, and transparent reporting of findings. Limitations include its single arm design, absence of a comparator, and reliance on self-reported history or implementation outcomes which were considered when interpreting the findings. Findings support feasibility among users who engaged. |
| Nazareth 2021 [50]† | NIH Observational Cohort and Cross-Sectional Studies | Fair | Large multicenter retrospective observational study with clear objective, defined population, high chatbot engagement, and well described uptake, completion, and genetic testing outcomes. A related article [11] provided context on the Gia chatbot’s clinical genetics workflow and genetic counselor involvement in content development. However, the study lacked a comparison group, relied partly on self-reported history, had missing data and platform/guideline changes, and did not capture genetic testing outside the platform. Findings support feasibility and scalability but should be interpreted cautiously. |
| Soley 2023 [15] | NIH Observational Cohort and Cross-Sectional Studies | Fair | Clear objective, defined pancreatic cancer population, and well-described Gia chatbot genetic education intervention were reported. However, this was a very small single-site feasibility study with an unclear eligible-participant denominator, no sample size justification, substantial survey missingness, limited demographic diversity, self-reported testing outcomes, and no confounder adjustment. Findings support preliminary feasibility only and should be interpreted cautiously. |
| Sato 2024 [43]  (quantitative component) | NIH Observational Cohort and Cross-Sectional Studies | Fair | Clear objective, defined clinical sample, standardized chatbot use, and comparison with information provided by certified genetic counselors were reported. However, the quantitative component was a very small single center pilot, and had limited ability to assess diagnostic accuracy or generalizability. Findings support feasibility only and should be interpreted cautiously. |
| Chavez-Yenter 2021 [45] | NIH Observational Cohort and Cross-Sectional Studies | Poor | Clear objective, defined electronic health record-identified primary care population, well-described chatbot pretest education intervention, and detailed transcript-based interaction outcomes were reported. A later BRIDGE analysis provided context on chatbot interaction patterns but did not alter the quality rating [14]. However, this study was small, had low completion rate among eligible patients, no sample size justification or control group, limited diversity, and no confounder adjustment. Findings support feasibility and user-interaction insights but should be interpreted cautiously. |
| Dohany 2020 [47]  (abstract) | NIH Observational Cohort and Cross-Sectional Studies | Poor | Clear objective and large multicenter ob/gyn implementation were reported, with chatbot completion and guideline-based risk criteria results described. However, as a conference abstract, methodological detail was limited; the eligible denominator was unclear, exclusions varied across practices, participant characteristics and algorithm validation were not fully reported, noncompletion/nonreceipt was high, and no confounder adjustment was performed. Findings provide descriptive feasibility context only and should be interpreted cautiously. |
| Monsour 2022 [49]  (abstract) | NIH Observational Cohort and Cross-Sectional Studies | Poor | Clear objective, rural gastroenterology clinic implementation, assessment completion rate, guideline-based risk criteria results, testing uptake, and genetic test outcomes were reported. However, as an abstract, methods were sparsely described; eligibility, implementation procedures, participant characteristics, algorithm/variant-classification details, and reasons many eligible patients were not tested were unclear. No confounder adjustment was performed. Findings provide descriptive implementation context only and should be interpreted cautiously. |
| Maisenbacher 2021 [48]  (abstract) | NIH Observational Cohort and Cross-Sectional Studies | Poor | Clear objective and chatbot assistant–based hereditary cancer risk assessment were reported, with completion and guideline-based risk criteria results described. However, as a conference abstract, methods were sparsely reported; the eligible denominator, recruitment procedures, participant characteristics, implementation consistency, algorithm validation, and data completeness were unclear. No sample size justification or confounder adjustment was provided. Findings provide descriptive feasibility context only and should be interpreted cautiously. |
| Al-Hilli 2023 [22] | RoB 2 | Some concerns | Randomized single-center trial with clear eligibility criteria, participant flow reporting, sample size rationale, and structured knowledge/satisfaction measures. However, allocation concealment was not fully described; the study was small and unblinded; satisfaction was self-reported; some outcome data were missing; and noninferiority reporting was limited. |
| Kaphingst 2024 [44] | RoB 2 | Some concerns | Multisite randomized equivalence trial with prespecified eligibility, site-stratified randomization, published protocol plan [20], objective service-use outcomes, and clear participant flow. However, many patients were excluded after randomization, participants and clinical teams were unblinded, and reach depended on electronic health record family-history data and patient portal access. Findings are internally strong but should be interpreted with these limitations. |

## † Initial discrepancy between two independent reviewers; resolved by consensus (final rating shown).

**Abbreviations:** CASP, Critical Appraisal Skills Programme; NIH, National Institutes of Health; RoB 2, revised Cochrane risk-of-bias tool for randomized trials.

**Note:** Related development, implementation, and protocol papers were consulted, when available, to clarify intervention context, chatbot design, or prespecified methods. These papers were not treated as independently included studies or as primary outcome evidence unless they were listed as included studies in the review.

For mixed-methods studies, qualitative and quantitative components were appraised separately using the most appropriate tool but were counted as a single included study in the review.
